# Simulation and Experiment on Droplet Formation and Separation for Needle-Type Micro-Liquid Jetting Dispenser

**DOI:** 10.3390/mi9070330

**Published:** 2018-06-29

**Authors:** Shizhou Lu, Guangyu Cao, Hai Zheng, Dongqi Li, Meiyan Shi, Jiahui Qi

**Affiliations:** 1 School of Mechanical, Electrical & Information Engineering, Shandong University at Weihai, Weihai 264209, China; zhenghai@mail.sdu.edu.cn (H.Z.); dq990301@mail.sdu.edu.cn (D.L.); smy_carrie@mail.sdu.edu.cn (M.S.); qijiahui@mail.sdu.edu.cn (J.Q.); 2 School of Mechatronics Engineering, Harbin Institute of Technology, Harbin 150001, China; cao_guangyu@mail.sdu.edu.cn

**Keywords:** needle-type, non-contact dispensing, droplet, piezoelectric actuator

## Abstract

The needle-type droplet jetting dispenser has wide applications in the field of microelectronic packaging, and for which the good quality of droplet formation and separation is the key to successful dispensing. This paper simulates the droplet jetting process which has been divided into 5 stages named backflow, growth, droplet extension, breakage, and separation, and analyses the combined effects of system parameters, such as pressure, viscosity, needle stroke, and nozzle diameter, on the changes of morphologies of ejected droplets, which is verified by experiments. The simulation and experiment results show that a higher driving pressure is quite suitable for the high-viscosity liquid to form normal droplets by avoiding adhesion. When increasing the needle stroke, the pressure should also be lowered properly to prevent the flow-stream. Besides, the nozzle with a large diameter is much more likely to cause sputtering or satellite-droplet problems. The results have a great significance for guiding the parameter settings of the needle-type dispensing approach.

## 1. Introduction

In the microelectronic packaging field, the dispensing of various adhesive liquids is an important technique to ensure the packaging quality of microelectronic chips [1,2,3]. In recent years, with the development of semiconductor technologies, many emerging packaging approaches, such as Ball Grid Array (BGA) and Chip Scale Package (CSP), have caused the size of chips to become smaller and the pin numbers to become greater [3,4,5]. In addition, many new adhesive materials are applied in the microelectronic packaging field to achieve the functions of filling, bonding, electrical or non-electrical connection [4,5]. In such circumstances, the micro-droplet dispensing technology is required to eject droplets with the characteristics of higher accuracy, smaller size, and faster speed.

At present, contact and non-contact are the main two kinds of dispensing techniques in production [6,7]. The contact type, with the features of large driving force and easy control, is widely used to dispense some high-viscosity adhesives [8,9]. However, this method must keep the dispensing nozzle close to the circuit board, resulting in low work efficiency, poor precision, and risk of liquid contact pollution, what makes it difficult to meet the requirements of microelectronic packaging field [8]. On the contrary, the non-contact dispensing method can eject the droplet directly by instantaneous high pressure without vertical motion [6]. In addition, the advantages of high dispensing efficiency, small droplet size and low requirement on operating space offers this method wide applications [6,8]. At present, a needle-type non-contact dispensing method, which utilizes a positive striking needle to drive the liquid jet out from the nozzle and form droplets, has a wide range of applications in the field of microelectronic packaging [10,11,12].

For the proposed needle-type non-contact dispensing method, the good quality of droplet formation and separation from the tip of nozzle is the key to successful dispensing. In addition, many researchers have been building many models of the proposed method to study the liquid dispensing process. For example, Ngyen et al. have built the equivalent mechanical model of a piezoelectric actuator-driven needle-type jet dispensing system according to the lumped parameter method and studied the effects of system parameters on needle vibration, liquid flow rate and volume [12,13,14]. Lu et al. have constructed the bond graph model of a needle-type micro-droplet dispenser featuring piezostack and diamond amplifying mechanism and studied the influence of the structure, driving pressure, frequency, and other control parameters on droplet flow rate and volume [6,15]. Deng et al. have established a simulation model of a needle-type droplet dispensing system and studied the influence of parameters such as driving pressure and needle stroke on droplet volume [16,17,18]. Liu et al. have simulated the distribution of pressure and velocity in dispensing chamber and studied the effects of the gap between the nozzle and the needle, the driving voltage, and the nozzle structure on the ejection performance [19,20]. Wang et al. have simulated and studied the influence of driving pressure and nozzle diameter on flow behavior according to a micro-liquid jetting valve with a hemispherical head [21].

The above research is of great significance in guiding the design and control of the proposed needle-type micro-droplet jetting systems. However, the detailed information of droplet formation and separation process is not yet clarified completely and in-depth research is still needed.

Focusing on the above problems and requirements, this paper simulated the changes in morphologies of droplet formation and separation, which have been divided into five stages named backflow, growth, extension, breakage, and separation, under the hitting effect of needle vibration. In the next step, the influences of system parameters such as pressure, needle stroke, and liquid viscosity on droplet jetting process were studied, and the causes of some abnormal dispensing results were also analyzed. Finally, an experiment system was established to verify the reliability of the simulation results and further study the influences of various parameters on droplet dispensing.

## 2. The Principle of Needle-Type Jetting Method and the Approach of Simulation

**Figure 1:**
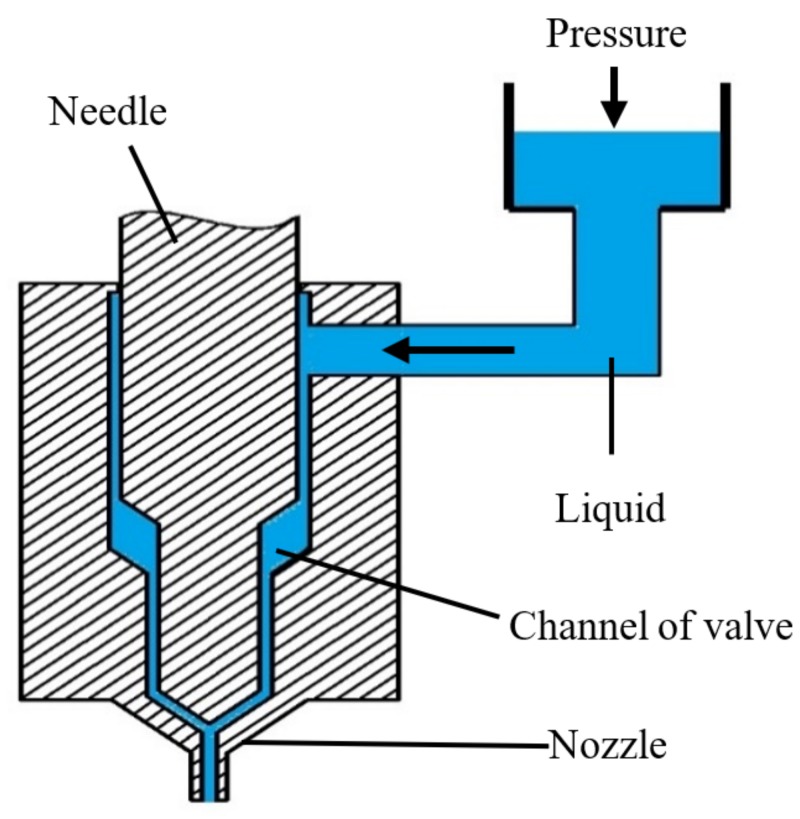
Principle of needle-driven type micro-injection.

The configuration of a needle-type non-contact droplet dispenser can be shown in Figure 1. According to this Figure, the droplet jetting process can be described thus: first, the needle is lifted by a piezoelectric actuator or an air-controlled valve, the pressured liquid fills the gap produced by needle motion, and flows out from the nozzle; then, when the needle moves down speedily and hits the nozzle, the flow will be blocked and the ejected liquid will be separated into droplets. Thus, under the continuous effect of needle vibration, a series of droplets with the desired volumes can be obtained.

In the paper, Fluent software is adopted to conduct the simulation works. The droplet separation from the nozzle into the air is a typical two-phase flow problem, so the volume of fluid (VOF) model was used for simulation. As the droplet shape changing process is always axisymmetric, the nozzle axis is parallel to the gravitational field and the needle vibrating direction, the flow in the nozzle can be processed on axial symmetry without circumferential velocity [22]. Thus, the droplet jetting process becomes a two-dimensional axisymmetric problem.

**Figure 2:**
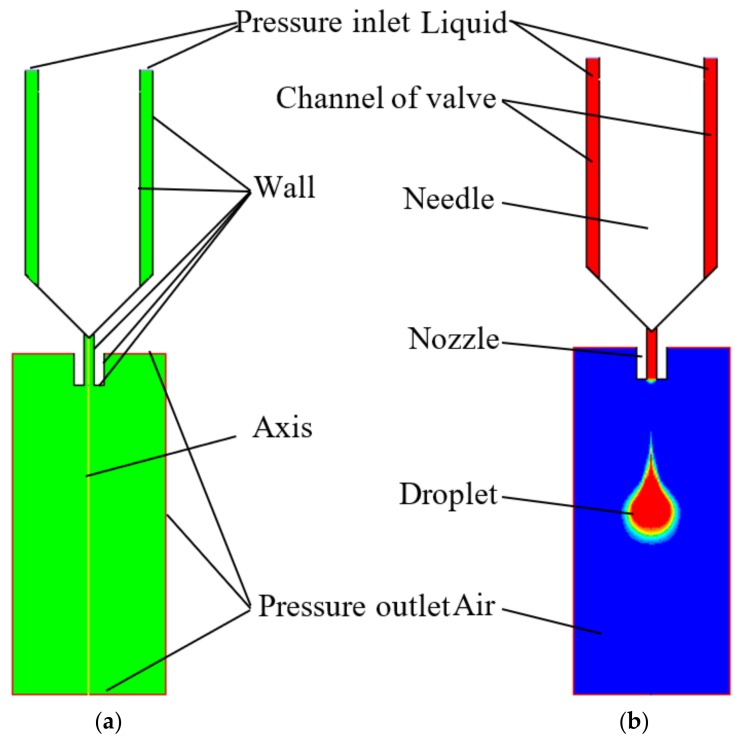
The simulation models: (**a**) gambit mesh model; (**b**) computational fluid dynamics (CFD) simulation model.

Gambit software is chosen to construct and mesh the physical model as Figure 2a shows. Where the upper surface of the liquid flowing into the nozzle is set as pressure inlet boundary condition, the gas interface is set as pressure outlet, the inner and outer walls and end plane of the nozzle are set as wall boundary conditions, the wall surfaces of the body and the needle are also set as wall boundary conditions, and the symmetry axis of the model adopts the axis boundary condition. After the model is imported into Fluent, its upper part is set to liquid and its lower part is set to air as Figure 2b shows. Since the reference pressure defaults to the standard atmospheric pressure, the pressure outlet of the gas interface is set to 0 MPa. In addition, the dynamic grid technology and the profiles of function are used to move the needle up and down. In the simulation process, parameters such as driving air pressure, liquid viscosity, nozzle diameter, and needle stroke are adjusted to obtain corresponding phase diagrams and animations of droplet micro-injection, which are used to conduct some further studies on droplet jetting mechanism.

The ranges of the simulation parameters in this paper are shown in Table 1.

## 3. Droplet Formation and Separation Analysis

**Figure 3:**
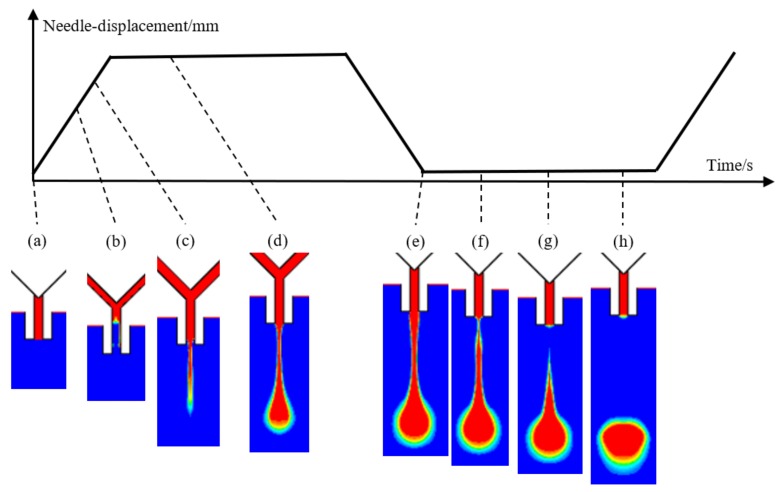
The droplet morphologies in a jetting cycle under the needle vibration: (**a**) start; (**b**) backflow; (**c**) growth; (**d**) extension; (**e**) maximum; (**f**) constriction; (**g**) breakage; (**h**) separation.

For the needle-type droplet jetting method, a trapezoidal wave is always chosen as the required vibration curve of needle as Figure 3 shows. According to this Figure, the droplet formation and separation process is simulated and divided into the following five stages:

(1) The backflow stage. Initially, the needle blocks the nozzle, and the channels of the dispenser are filled with adhesives. At the same time, the liquid in the nozzle has a concave surface due to the action of surface tension and capillary force as Figure 3a shows. When the needle moves up, the generated negative pressure zone in the channel causes the liquid to flow back from the nozzle as Figure 3b shows.
(2) Droplet growth stage. With the continuous lifting of the needle, the high pressure affecting the adhesives in syringe drives the liquid to move along the channel and flow out from the nozzle. During this process, the original concave surface extends to form a new convex shape with the appearance of liquid column under the nozzle tip as shown in Figure 3c.
(3) Droplet extension stage. The fluid column becomes longer with the volume increase of liquid flowing out form the nozzle (Figure 3d) and reaches the biggest size before the needle moves down and blocks the nozzle again (Figure 3e). From then, no new liquid is added to the droplets.
(4) Droplet breakage stage. The flow rate of liquid inside the nozzle drops to zero due to the block between the nozzle and the upper channel. The outside part still has a downward thrust to elongate the liquid column further because of inertia action. In addition, the difference in speed makes the radius of the liquid column below the nozzle smaller (Figure 3f) and separates the column into two parts (Figure 3g).
(5) Droplet separation stage. The tapered tail of the ejected liquid moves speedily to the main body of droplet because of surface tension, which makes the droplet have a better ball shape and leave the nozzle finally (Figure 3h). In addition, the liquid remaining at the end of the nozzle is sucked back to form a new inward concave surface again as Figure 3a shows.

**Figure 4:**
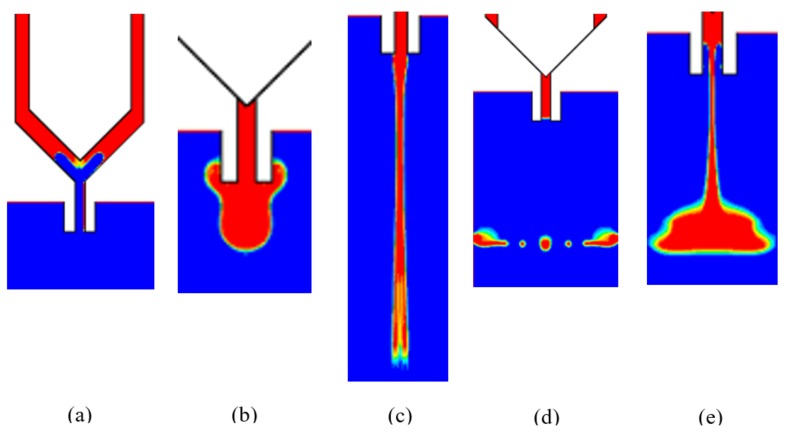
Dispense failure situation: (**a**) excessive back suction; (**b**) adhesion; (**c**) flow-stream; (**d**) satellite droplets; (**e**) sputtering.

As can be seen from the above analysis, the needle vibration and the pressure drive achieve the droplet formation and separation, and a successful jetting process should at least satisfy the following conditions: (1) In the backflow stage, the amount of liquid flowing back into the channel should be controlled in a suitable range, otherwise some bubbles may be mixed within the droplet, which might be broken into lots of scattered dots (Figure 4a); (2) During the droplet growth stage, no ejected liquid remains on the nozzle tip, otherwise the created thick liquid layer will stop the flow (Figure 4b); (3) During the extension and breakage stage, the jetting speed should also be in a suitable range, otherwise a long stream or satellite dots might appear as Figure 4c–e show. The above abnormal jetting situations are related to the system parameter settings. To effectively guide the droplet micro-jetting process and ensure stable droplet separation, the paper will further study the effects of driving and structural parameters such as air pressure, viscosity, needle stroke and nozzle diameter on the changes in morphologies of droplets.

## 4. The Influences of System Parameters on Droplet Dispensing

### 4.1. The Influences of Viscosity and Pressure on Droplet Dispensing

First, the paper studies the effects of viscosity and pressure on droplet formation and separation process, during which the other related parameters are set thus: the surface tension is 0.03 N/m, the nozzle diameter is 0.2 mm, the needle stroke is 0.3 mm.

**Figure 5:**
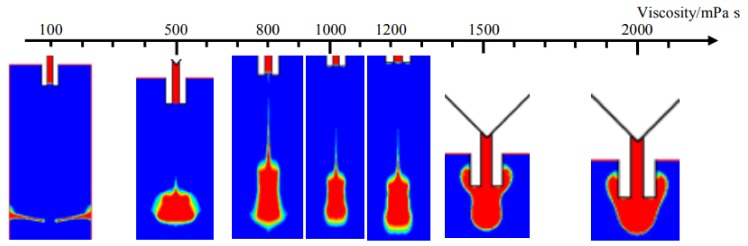
Simulation results from changes in viscosity.

As shown in Figure 5, the droplet jetting results are demonstrated when the pressure is 0.7 MPa and the viscosity changes in the range of 100 to 2000 mPa·s. It can be seen from Figure 5 that it will be more difficult to form a stable droplet with the increase of liquid viscosity due to the increased viscous resistance. When using a high pressure to dispense a low-viscosity liquid, the droplet carrying overfull energy can break through the restriction of viscous force quite easily and scatter into several smaller dots after jetting out from the nozzle. When the viscosity increases to a suitable range matching the applied pressure, the expected droplet can be obtained. However, the continuing increased viscosity will lower the narrow position during the breakage stage and cause more liquid to remain on the nozzle tip after separation. Even worse, the strong viscous force will stop the flow completely and make the liquid just adhere to the nozzle due to the high viscosity.

**Figure 6:**
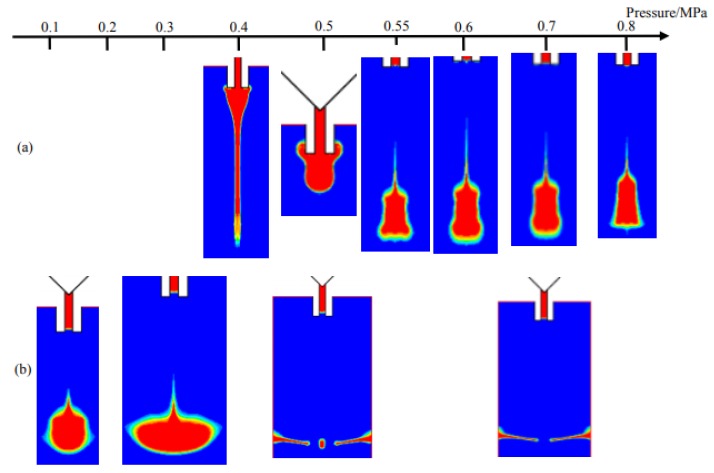
Simulation results from changes in pressure when viscosity is (**a**) 1000 mPa·s; (**b**) 100 mPa·s.

Two groups of simulation are undertaken when the pressure is within 0.1–0.8 MPa, the viscosities are set to 1000 mPa·s and 100 mPa·s respectively as Figure 6 shows. As depicted in Figure 6a, it is much easier to form stable high-viscosity droplets with the increase of pressure. In addition, in Figure 6b, the changes from normal ejection to satellite dots and then sputtering appear due to the increased energy in droplets.

**Figure 7:**
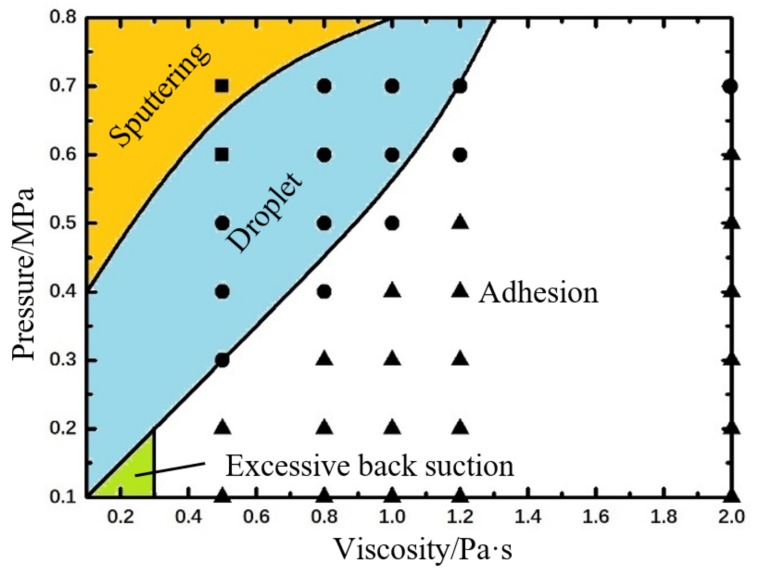
The combined effects of pressure and viscosity on droplet formation and separation process based on numerical simulations. The symbols, “●” indicates droplet, “▲” indicates adhesion, “■” indicates sputtering, represent experimental results. The experimental parameters are set as: nozzle diameter: 0.2 mm, length: 2 mm, needle stroke: 0.3 mm, frequency: 20 Hz.

In the next step, according to the simulation results shown in Figure 5 and Figure 6, the comprehensive influences of viscosity and pressure on droplet formation and separation are shown in Figure 7. As demonstrated in this Figure, the sputtering phenomenon is mainly caused by the high pressure and low viscosity. Under this circumstance, the droplet can get excessive kinetic energy before breaks the weak viscous resistance, resulting in sputtering or satellite dots. Besides, when the pressure is also in a low range, more liquid mixed with air-bubbles may flow back to the upper channels during the backflow stage. With the continuing increase of viscosity, the liquid flowing out from the nozzle needs to overcome the large viscous restriction, which leads to adhesion phenomenon. Only when the liquid viscosity and driving pressure are both in the suitable range, the normal droplet dispensing process can be obtained.

### 4.2. The Influence of Pressure and Needle Stroke on Droplet Dispensing

In this part, the combined effects of pressure and needle stroke on droplet formation and separation are simulated. The needle motion is controlled by the function which is written in the text document. In addition, the other related parameters are set thus: the surface tension is 0.06 N/m, the nozzle diameter is 0.2 mm, the viscosity is 800 mPa·s.

**Figure 8:**
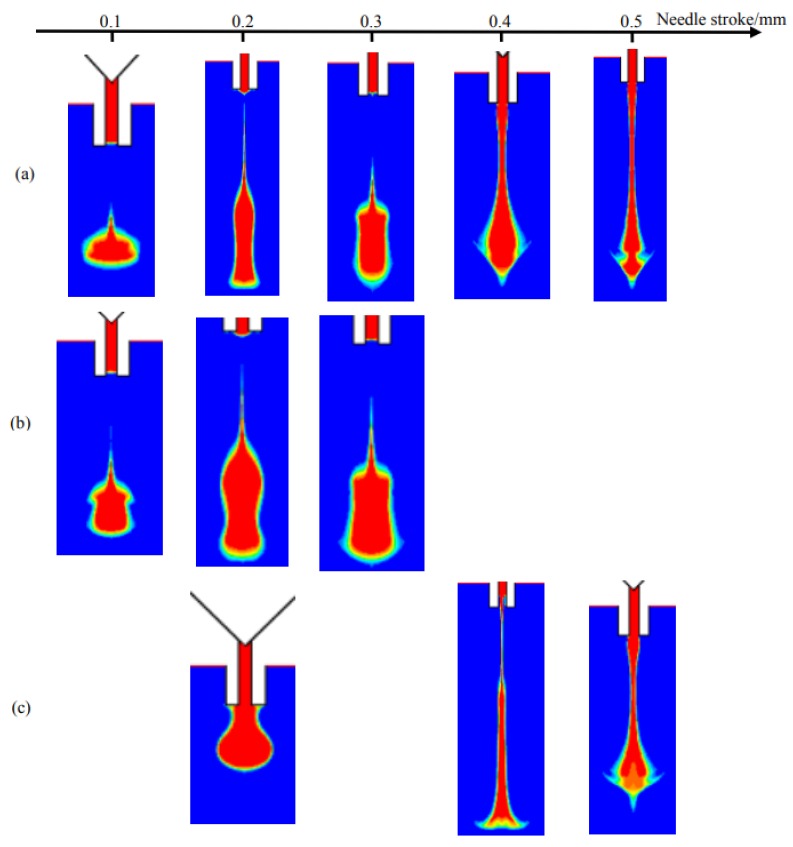
Simulation results from the changes in needle stroke when pressure is (**a**) 0.6 MPa; (**b**) 0.8 MPa; (**c**) 0.3 MPa.

Figure 8 shows the droplet morphologies obtained at different pressure and needle stroke conditions. As depicted in Figure 8a,b, more liquid will flow out from the nozzle with the increase of needle stroke due to the decreased flow resistance and the expanded gap between the nozzle and the upper channel. When the stroke is within 0.3 mm, the normal droplets can be obtained easily. However, the fluid-streams with too much liquid than normal droplet will appear when the stroke is larger than 0.4 mm. It can be predictable that a higher-viscosity liquid requires a rising feasible range of needle stroke because of the increased viscosity resistance. As shown in Figure 8c, when reducing the pressure, the liquid may cannot jet out from the nozzle due to the low driving force but high flow resistance. Furthermore, when the needle stroke increases to 0.4 mm, the liquid stretches too long and falls on the circuit due to gravity.

**Figure 9:**
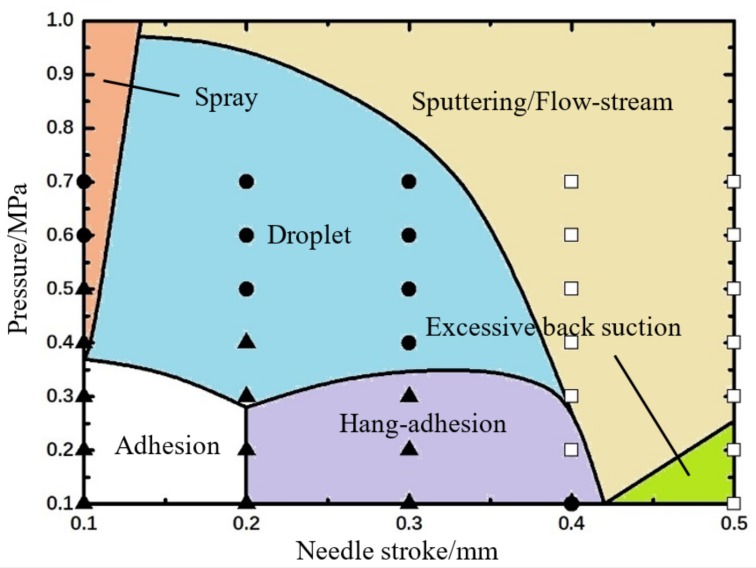
The combined effects of pressure and needle stroke on droplet formation and separation process based on numerical simulations. The symbols represent experimental results: “●” indicates droplet, “▲” indicates adhesion, “□” indicates flow-stream. The experimental parameters are set as: viscosity: 800 mPa·s, nozzle diameter: 0.2 mm, length: 2 mm, needle cycle: 20 Hz.

According to the above simulation results, the combined effects of pressure and needle stroke on droplet micro-jetting are obtained as Figure 9 shows.

As shown in Figure 9, the quite low pressure and stroke will make the liquid just remains on the nozzle tip. That is because little energy can be provided for the liquid to break the excess resistance of flow or viscosity. However, the liquid spray will appear if only increasing the pressure to a large value. In addition, if just increasing the needle stroke, more liquid will hang at the nozzle and form a long fluid line before falling. What is worse, the over-long stroke might cause the air flow into the upper channels during the backflow stage. On the other hand, the over-large pressure can provide excessive energy for the droplets resulting in sputtering or flow-stream. Only when the driving pressure and the needle stroke are both in the suitable rage, the droplet formation and separation process can be performed normally. In addition, the maximum pressure required to achieve normal ejection also gradually decreases with the increase of stroke.

### 4.3. The Influences of Nozzle Diameter and Needle Stroke on Droplet Dispensing

The results from the simulation and experiment indicate that the nozzle can also affect the dispensing greatly. Thus, the combined effects of nozzle diameter and needle stroke on droplet formation and separation are simulated. The nozzle models with different diameters have been built by Gambit before simulation, and the other related parameters are set thus: the surface tension is 0.06 N/m, the driving pressure is 0.6 MPa, the viscosity is 800 mPa·s.

**Figure 10:**
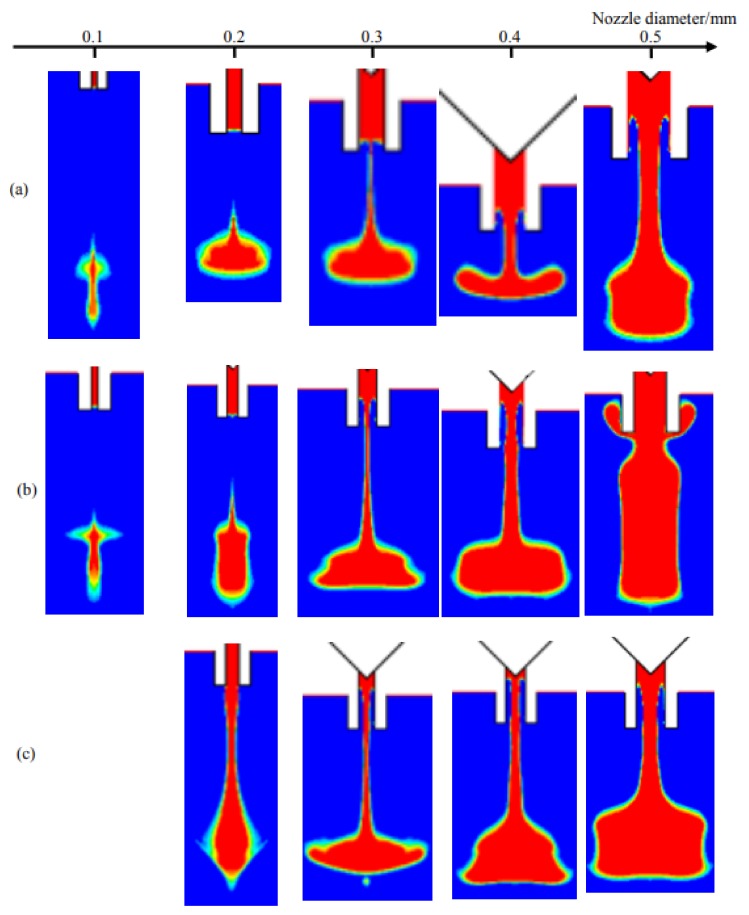
Simulation results from changes in nozzle diameter when stroke is (**a**) 0.1 mm; (**b**) 0.3 mm; (**c**) 0.4 mm.

**Figure 11:**
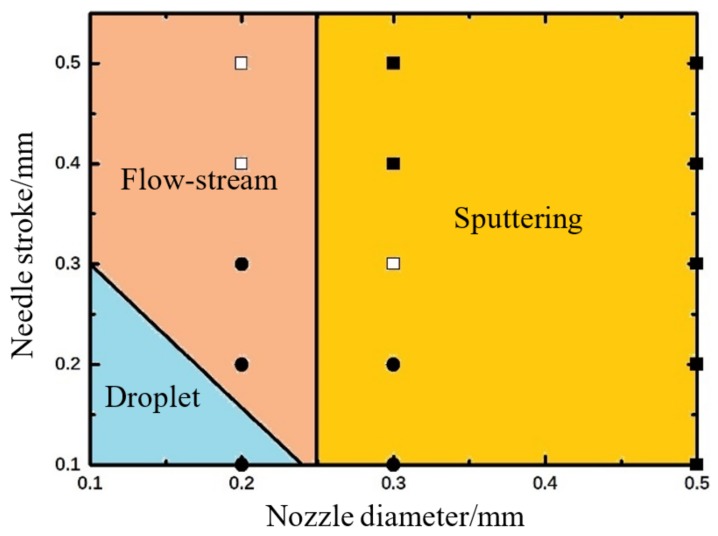
The combined effects of nozzle diameter and needle stroke on droplet formation and separation process based on numerical simulations. The symbols represent experimental results: “●” indicates droplet, “□” indicates flow-stream, “■” indicates sputtering. The experimental parameters are set as: viscosity: 800 mPa·s, nozzle length: 2 mm, driving pressure: 0.6 MPa, needle cycle: 20 Hz.

Figure 10 shows the droplet morphologies obtained at different nozzle diameter and needle stroke conditions. It can be clearly seen that more adhesives will flow out with the increase of nozzle diameter. As we know, the nozzle diameter directly relates to the flow rate and resistance. When the diameter is just 0.1 mm, the small flow rate and big resistance lead to fewer liquid jetting out from the nozzle in the form of micro dots as Figure 10a,b show. As the diameter increases to 0.2 mm, more liquid can be ejected as droplet. However, the continuing increase of diameter will enlarge the flow rate and reduce the flow resistance greatly, which makes an amount of liquid carrying excessive energies flow out and hang at the nozzle tip in a long streamline. Even worse, the narrow position of the streamline will be lifted into the inner part of nozzle by resulting in uncontrolled volume and failure of the following dispensing cycle. It can also be found that a higher needle stroke will worsen the problems mentioned above. Thus, the combined effects of nozzle diameter and needle stroke are depicted in Figure 11.

As Figure 11 shows, the nozzle with a bigger diameter needs a smaller stroke to ensure normal droplet jetting. The raising of any parameter will lead to flow or sputtering phenomenon. Thus, these two factors should be controlled in the triangle region during parameter setting process.

## 5. Experiment Results and Analysis

**Figure 12:**
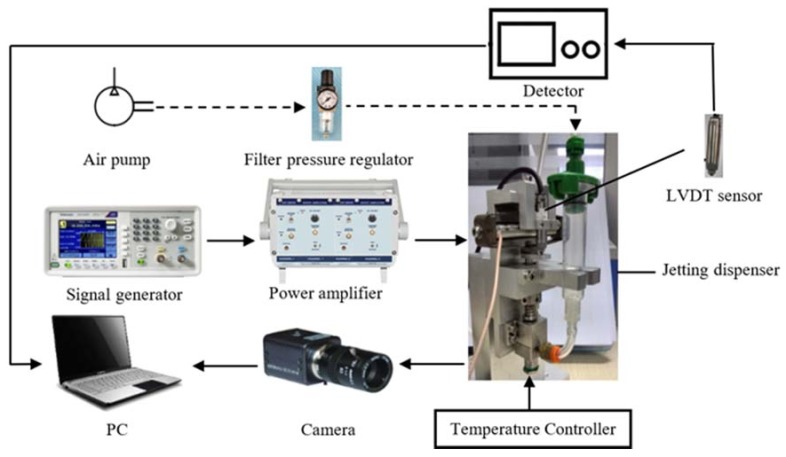
The schematic of the jetting experimental system.

**Figure 13:**
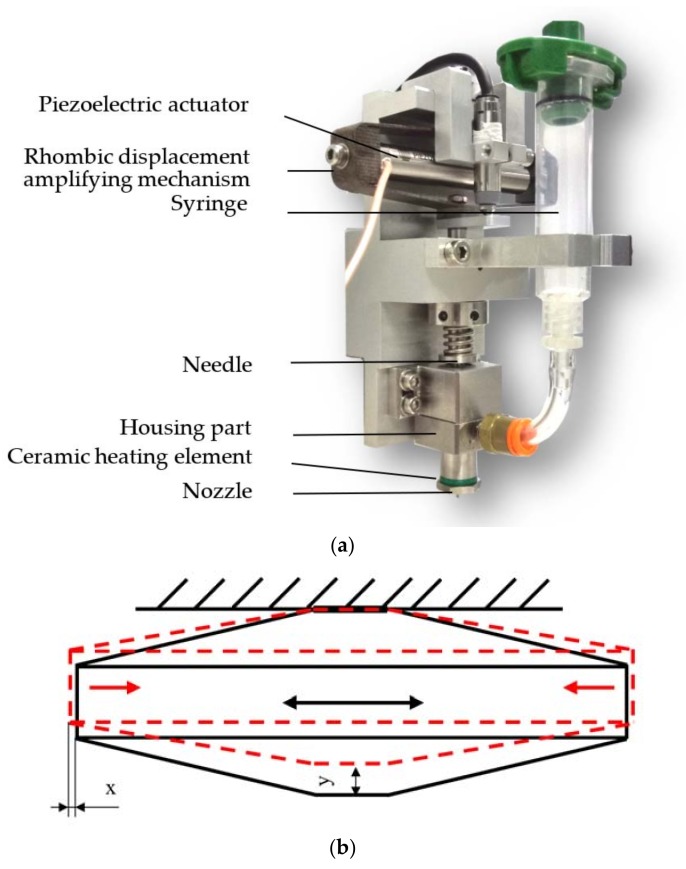
(**a**) Jetting dispenser; (**b**) Rhombic displacement amplifying mechanism; the black Figure is the state when the piezoelectric actuator is not energized, and the red figure is the state when the piezoelectric actuator is energized; “x” is half of the displacement produced by this piezoelectric actuator; “y” is the stroke of needle.

**Figure 14:**
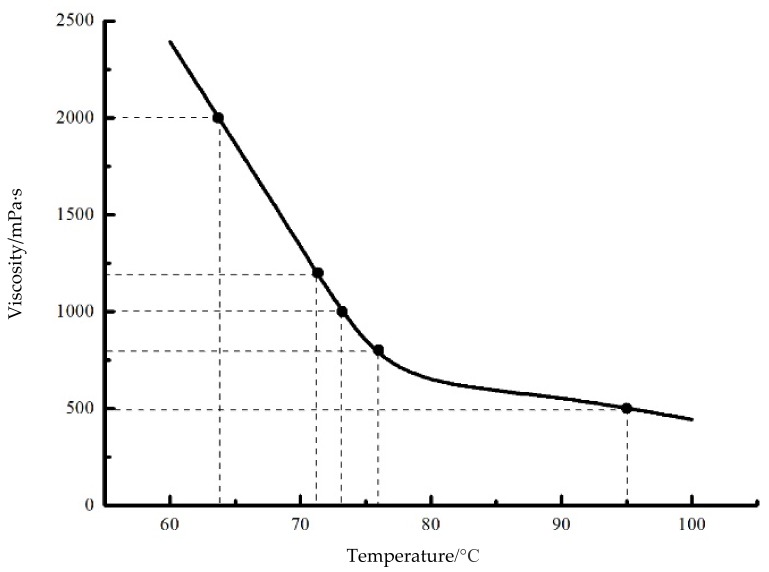
Viscosity and temperature characteristic curve of the epoxy glue.

To verify the simulation results and further study the droplet formation and separation mechanism, an experimental platform is adopted as shown in Figure 12. As this figure shows, the experiment platform mainly consists of a needle-type piezoelectric actuator driven dispenser, a function signal generator (AFG1062, Tektronix, Beaverton, OR, USA), a power amplifying module (E01.A2, Coremorrow, Harbin, China), an assembled pneumatic control unit, a linear variable differential transformer (LVDT) sensor (SL1500, AMETEK, Berwyn, PA, USA), and a camera (Manta G-223B, Allied Vision Technologies, Stadtroda, Germany). As Figure 13a shows, the proposed jetting dispenser is mainly composed of a piezoelectric actuator (PST150/5/60, Piezomechanik, Münich, Germany), a rhombic displacement amplifying mechanism, a housing part, a syringe, a needle, a nozzle, and a ceramic heating element. It should be noted that the maximum displacement produced by this piezoelectric actuator is only 60 micrometers, which cannot meet the requirement of the dispenser. Thus, the mentioned rhombic mechanism, whose internal part and vertical end connect to the piezoelectric actuator and needle top respectively, is adopted to magnify the displacement of piezoelectric actuator by about ten times before converting it into the vertical stroke of needle vibration as Figure 13b shows. The syringe is used to contain the adhesive whose viscosity will be adjusted by the heating element. The camera is used to capture the droplet images during the experiment. In the working process, as the amplified voltage signal functions on the piezoelectric actuator, the needle will be lifted and lowered under the driven of rhombic mechanism, and the stroke will be tested by the LVDT sensor. The pressured adhesive containing in the liquid syringe can be driven into the housing part of the dispenser by the pneumatic control unit and ejected out from the nozzle to form droplet by the hitting of needle vibration. In this paper, an epoxy glue (WQ-M030, Yihui, Dongguan, China) is adopted during experiment and its viscosity under different temperature is shown in Figure 14.

During the experiments, the air-pressure applied to the adhesive can be changed by the filter of the pneumatic control unit, the viscosity of the epoxy glue can be controlled by the heating element, the stroke and vibration frequency of the needle can be adjusted by changing the amplitude and frequency of the signal which is generated by the function signal generator and power amplifying module. Thus, by changing the above parameters, different experimental conditions can be obtained.

**Figure 15:**
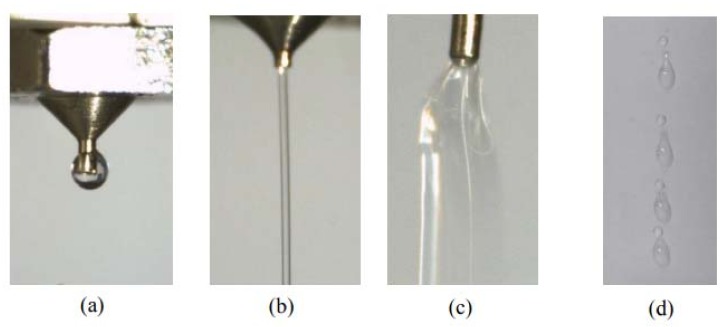
Jetting failure situations: (**a**) adhesion; (**b**) flow-stream; (**c**) sputtering; (**d**) satellite droplets.

In the next step, the several jetting failure situations, such as adhesion, flow-stream, sputtering, and satellite dots, are pictured, as Figure 15 shows. Furthermore, several symbols are used to describe the different dispensing states: “●” indicates a stable droplet ejection process (Droplet); “▲” indicates that the droplet adheres to the nozzle (Adhesion); “□” indicates the long flow-stream under the nozzle tip; “■” indicates Sputtering or Satellite Droplets. Then, the relevant experimental conditions and results are shown in Figure 7, Figure 9 and Figure 11.

As the experimental results in Figure 7 show, the increase of viscosity will change the droplet status from sputtering to droplet and then to adhesion. However, the pressure depicts an opposite effect trend. The sputtering occurs when the viscosity is relatively small and the driving force is large. When the viscosity is relatively large and the driving force is small, the adhesion phenomenon occurs, which means a higher pressure is required to provide enough energy to overcome the viscous resistance. Through the above analysis, it can be clearly seen that the experiments match the simulation results very well.

Figure 9 shows the experiment results according to different driving pressures and needle strokes. It can be demonstrated from this Figure that the adhesion situation occurs when the pressure and stroke are both in a small range. When the stroke is smaller than 0.4 mm, the increase of pressure would produce normal ejected droplets. However, if the stroke becomes larger, the flow-stream status will appear even when the pressure is in a quite small value. In addition, this control advice obtained from the experimental results agrees with the simulation results.

Figure 11 shows the experiment results according to different nozzle diameters and needle strokes. As it shows, the normal dispensing status can be obtained when the stroke and diameter are both in small ranges. Otherwise, the increase of any factor will cause sputtering or satellite-droplet problems, which also can be found in the simulation results in Figure 11.

## 6. Conclusions

This paper has simulated the droplet formation and separation process on a needle-type non-contact micro-liquid dispensing approach, and analyzed the combined effects of system parameters, such as pressure, viscosity, needle stroke, and nozzle diameter, on the changes of morphologies of ejected droplets. The mentioned process has been divided into 5 stages named backflow, growth, droplet extension, breakage, and separation, and during each stage the possible failed jetting matters, such as adhesion, sputtering, flow-stream, or satellite dots, were also discussed. Finally, an experiment platform containing a needle-type piezoelectric actuator-driven dispenser was built, and the experiments verified the reliability of the simulation results. From the experiment and simulation results, the paper finds that a higher driving pressure is quite suitable for the high viscosity liquid to form normal droplets by avoiding adhesion. However, when increasing the needle stroke, the pressure should be lowered appropriately to prevent the flow-stream. Furthermore, a nozzle with a larger diameter is much easier to cause sputtering or satellite-droplet problems. The results are used to guide the design and control works of these kinds of needle-type jetting dispensers. In further study, research regarding droplet volume (especially the minimum value of volume obtained under different jetting conditions), and duty cycle influence during the continuous injection process should be undertaken, and some effective measures to improve precision for this kind of dispenser should also be explored deeply.

## Figures and Tables

**Table 1:** The ranges of simulation parameters.

Driving Pressure (MPa)	Liquid Viscosity (mPa·s)	Needle Stroke (mm)	Nozzle Diameter (mm)	Liquid Density (kg/m^3^)	Surface Tension (N/m)	Nozzle Length (mm)	Needle Cycle (Hz)
0.1–0.8	100–2000	0.1–0.5	0.1–0.5	1200	0.03–0.06	2	20
